# Supplementary material for: Splice-Junction-Based Mapping of Alternative Isoforms in the Human Proteome
Source: Cell Rep. Author manuscript; Available in PMC 2020 Jan 15. (PMC6961840; doi:10.1016/j.celrep.2019.11.026)
Supplement: 2 [file NIHMS1546469-supplement-2.zip › DF1/PXD009737/TestisGluC/TestisGluC_3_PPP4R1_RCFVSDRTISLQQATQYSEQKIGT.pdf]

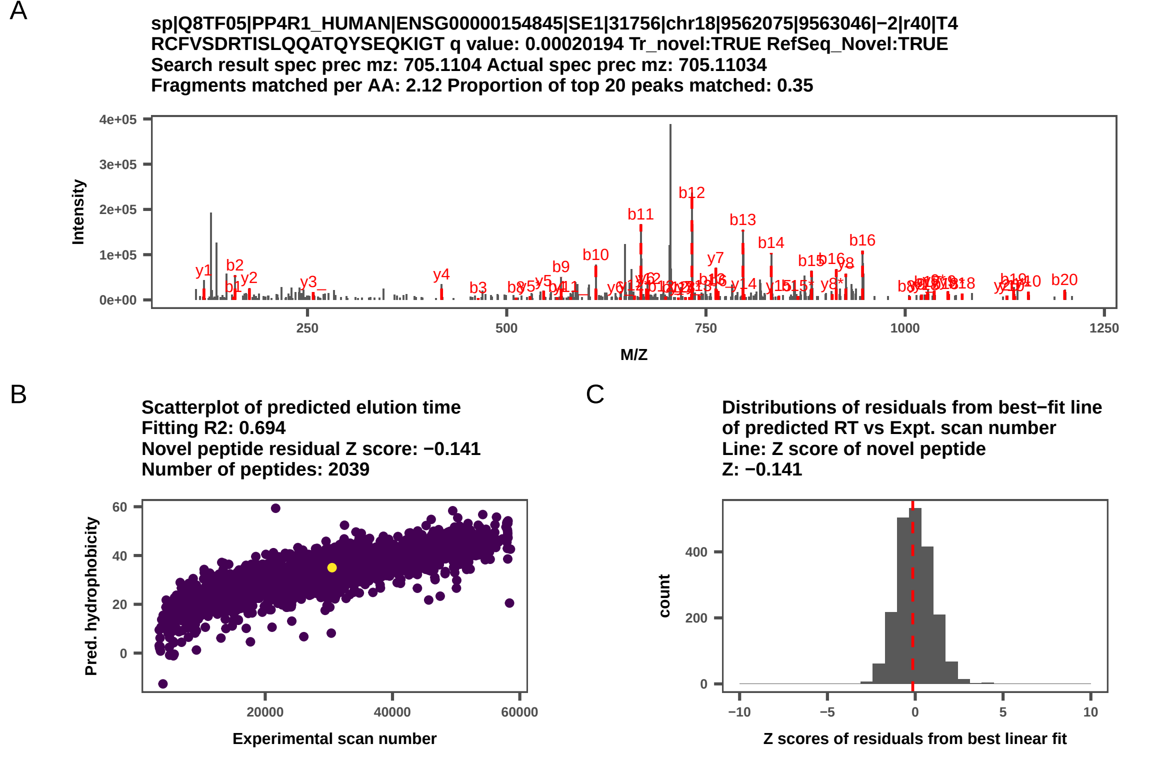
A
sp|Q8TF05|PP4R1_HUMAN|ENSG00000154845|SE1|31756|chr18|9562075|9563046|−2|r40|T4
RCFVSDRTISLQQATQYSEQKIGT q value: 0.00020194 Tr_novel:TRUE RefSeq_Novel:TRUE
Search result spec prec mz: 705.1104 Actual spec prec mz: 705.11034
Fragments matched per AA: 2.12 Proportion of top 20 peaks matched: 0.35
Intensity
4e+05
3e+05
2e+05
1e+05
0e+00
y1
b2
b1
y2
y3_
y4
b3
b9
b10
b11
b12
b13
b14
b16
y7
y8
y14
b20
250
500
750
1000
1250
M/Z
B
Scatterplot of predicted elution time
Fitting R2: 0.694
Novel peptide residual Z score: −0.141
Number of peptides: 2039
Pred. hydrophobicity
60
40
20
0
20000
40000
60000
Experimental scan number
C
Distributions of residuals from best−fit line
of predicted RT vs Expt. scan number
Line: Z score of novel peptide
Z: −0.141
count
400
200
0
−10
−5
0
5
10
Z scores of residuals from best linear fit
